# Clinical and cost-effectiveness of oral versus intramuscular glucocorticoids in rheumatoid arthritis: protocol for a multicentre randomised controlled trial with economic evaluation and qualitative sub-study (LEADER trial)

**DOI:** 10.1136/bmjopen-2026-119885

**Published:** 2026-07-10

**Authors:** Jonathan Alistair Cook, Nicky Wilson, Kate Wilkins, Karen Staniland, Iniobong Ekang, Shazli Azmi, Liam Heaney, Sean P Gavan, Rachel Ann Elliott, Robert J Wilson, Clare Calvert, Anne Francis, Jennifer Anne Elizabeth Williams, Anabella Turner, Alex Zimmermann, Duncan Richards, Anne Barton, James Bluett, The LEADER trial network, Hanu Reddy

**Affiliations:** 1 Oxford Clinical Trials Research Unit, Centre for Statistics in Medicine, Nuffield Department of Orthopaedics, Rheumatology and Musculoskeletal Sciences (NDORMS), University of Oxford, Oxford, England, UK; 2 Department of Rheumatology, King’s College Hospital NHS Foundation Trust, London, UK; 3 Centre for Rheumatic Diseases, Kings College London, London, England, UK; 4 Centre for Genetics and Genomics, Centre for Musculoskeletal Research, Division of Musculoskeletal & Dermatological Sciences, The University of Manchester, Manchester, UK; 5 Diabetes, Endocrinology and Metabolism, Manchester University NHS Foundation Trust, Manchester, Division of Cardiovascular Sciences, Faculty of Biology, Medicine and Health, The University of Manchester, Manchester, England, UK; 6 Wellcome-Wolfson Institute for Experimental Medicine School of Medicine, Dentistry and Biomedical Sciences, Queen's University Belfast, Belfast, Northern Ireland, UK; 7 Manchester Centre for Health Economics, Division of Population Health, Health Services Research and Primary Care, School of Health Sciences, Faculty of Biology, Medicine and Health, The University of Manchester, Manchester, UK; 8 Department of Rheumatology, University College London Hospitals NHS Foundation Trust, London, UK; 9 Oxford Clinical Trials Research Unit, Nuffield Department of Orthopaedics, Rheumatology, and Musculoskeletal Sciences (NDORMS), University of Oxford, Oxford, UK; 10 Department of Medicine, University of Cambridge, Cambridge, UK; 11 NIHR Manchester Biomedical Research Centre, Manchester University NHS Foundation Trust, Manchester, England, UK; 12 Airdale NHS Foundation Trust, Keighley, UK

**Keywords:** Randomized Controlled Trial, RHEUMATOLOGY, Treatment Outcome

## Abstract

**Introduction:**

Glucocorticoid (GC) bridging therapy is recommended in patients with rheumatoid arthritis commencing a disease-modifying anti-rheumatic drug (DMARD). It is not clear whether GC therapy is better administered intramuscularly or orally and at what dose level. The aim of the LEADER trial is to identify the most effective and safest way of using steroids in patients with uncontrolled RA who are starting a DMARD.

**Methods and analysis:**

A multicentre, randomised, open-label, four-arm, parallel-group clinical trial with an internal pilot phase, economic evaluation and qualitative study of acceptability. Participants will be randomised to one of four arms: arm A, 30 mg oral prednisolone tapering over 6 weeks; arm B, 15 mg oral prednisolone tapering over 4 weeks; arm C, Intramuscularly 120 mg methylprednisolone; and arm D, Intramuscularly 80 mg methylprednisolone. Participants will be assessed at baseline (pre-GC intervention), 4, 12 and 24 weeks. The primary outcome measure is the mean DAS(CRP)−28 over 12 weeks. The primary comparison will be according to route of administration (oral vs intramuscular GC treatment) with secondary comparisons within route of administration to provide evidence of dose effectiveness. Toxicity will be measured using the Glucocorticoid Toxicity Index, a clinical outcome assessment and early morning cortisol level. LEADER will be conducted in ~30 sites delivering NHS care, recruiting a sample size of 448. Economic evaluation will compare cost-effectiveness within a trial and over a lifetime horizon from the English National Health Service perspective.

**Ethics and dissemination:**

The LEADER trial received MHRA and Leicester Central Research Ethics Committee ethics approval (REC reference: 24/EM/0277, IRAS 1010280), opened to recruitment on Protocol Version 4.0 and is currently recruiting on Protocol Version 5.0. Participants will provide written informed consent in accordance with the Declaration of Helsinki and applicable regulatory requirements. Trial results will be disseminated via presentations at national and international meetings, published in open-access journals and to patients.

**Trial registration number:**

ISRCTN32090559.

**STRENGTHS AND LIMITATIONS OF THIS STUDY**

- Robust study design with adequate power to investigate the efficacy of oral versus intramuscular glucocorticoid (GC) bridging therapy in patients with rheumatoid arthritis (RA) initiating, escalating or switching disease-modifying anti-rheumatic drug therapy and secondary comparisons to provide evidence for GC dosage
- The trial will explore participant views on the acceptability of GC bridging therapy
- LEADER will provide a comprehensive within-trial and lifetime-horizon economic evaluation of intramuscular versus oral GC bridging therapy in RA incorporating both toxicity and effectiveness.
- Limitations include the requirement for further GC therapy for participants who flare during the trial period and voluntary blinding of DAS(CRP)−28 assessment.

## Introduction

 Rheumatoid arthritis (RA) is a chronic autoimmune joint disease affecting up to 1% of adults.^1^ The aim of RA treatment is remission using a treat-to-target approach of close and regular assessment of disease activity and timely therapy escalation.^2^ Rapid target attainment reduces radiographic progression and disability,^3^ but conventional disease-modifying anti-rheumatic drugs (cDMARDs) can take 6 months to improve disease activity^4 5^; glucocorticoid (GC) ‘bridging’ therapy is therefore recommended. A Cochrane review revealed that GCs reduce radiographic progression, joint tenderness, swelling and pain and improve physical functioning compared with placebo.^6^ GCs have a rapid onset of action but are associated with significant adverse effects. British and European guidelines recommend short-term (<3 months) GC therapy in patients commencing cDMARDs.^7 8^ International consensus about the use of GC bridging therapy in patients with RA has not been reached. Concerns about toxicity led the 2021 American College of Rheumatology Guideline to make a conditional recommendation, based on expert opinion, against bridging GC therapy.^9^ GCs can be administered orally (PO) or intramuscularly, but no guidelines exist recommending a preferred route of GC therapy. It remains unknown whether the route of administration, dose level or duration of GCs makes a meaningful difference for patients commencing a conventional, biologic or targeted synthetic DMARD.

Real-world observational studies have shown that GCs are commonly used and accepted by patients with RA and treating physicians. Within England and Wales, where national guidelines recommend GC bridging therapy, 76% of people diagnosed with RA were treated with GC therapy at the time they were given a working diagnosis, rising to over 82% within the first 6 weeks.^10^

Two clinical trials provide evidence for GC therapy during DMARD initiation. The tREACH trial was a randomised controlled trial of patients with early RA (symptoms <1 year) who were randomised to (1) methotrexate (MTX) + sulfasalazine (SSZ) + hydroxychloroquine (HCQ) (combination DMARD therapy) with intramuscular methylprednisolone 120 mg or triamcinolone 80 mg; (2) Combination DMARD therapy with oral prednisolone 15 mg/day tapered to 2.5 mg over 10 weeks and (3) MTX monotherapy with oral GC bridging similar to (2).^11^ In the combination arms at 3 months there was no significant difference in disease activity score (DAS) between the GC bridging therapies (change in DAS 0.03 95% CI −0.24 to 0.31). Earlier disease activity and GC toxicity were not captured, however. The CareRA trial investigated the effect of bridging GC therapy in patients with early disease duration (≤ 1 year) without poor prognostic features^12^ who were randomised to receive (1) MTX with step-up of dose (MTX-TSU) and no oral GC therapy or (2) MTX with 30 mg prednisolone tapered to 5 mg daily by week 6 (COBRA-slim). At week 16 there was no significant difference in mean DAS(CRP)−28 change in either group (MTX-TSU 1.76±SD 1.68 vs COBRA slim 2.12±SD 1.41); however, patients in the COBRA-slim arm experienced early disease control from week four which was sustained during GC tapering compared with the MTX-TSU arm, which did not receive GC therapy.

One of the major concerns of oral GC therapy is risk of toxicity and propensity for extended use. It is not yet known whether this risk is similar for patients who receive initial intramuscular GC bridging therapy. Patients with RA have an increased risk of GC-related adverse drug reactions (ADRs) such as diabetes (OR_adj_ 1.33), osteoporosis (OR_adj_ 1.41), stroke or myocardial infarction (OR_adj_ 1.28), serious infection (OR_adj_ 1.28) and death (OR_adj_ 1.3) where risk was estimated using matched controls.^13^ While the risk of ADRs is dose-dependent and time-dependent,^13^ low-dose prednisolone (7.5 mg daily) is also associated with complications such as hypertension in patients with RA.^14^ Results from a meta-analysis of clinical trials of oral GC bridging therapy showed that patients receiving oral bridging therapy were over three times more likely to remain on steroids by 12 months compared with patients who did not receive bridging treatment (OR 3.27)^15^ and recent real-world data have reported sustained use of steroids even in patients in remission.^16^

There is a clear knowledge gap regarding the optimal initial dose, tapering schedule and treatment duration of bridging GC in patients with RA that would offer superior outcomes while limiting toxicity.^17^ The clinical and cost-effectiveness of ora*l* v*s* intr*A*muscular glucocorticoi*D*s in rh*E*umatoid a*R*thritis (LEADER) trial aims to identify the most effective and safest way of using GCs in patients with uncontrolled RA who are starting a DMARD. The trial will assess whether an intramuscular injection or daily tablet regimen is better at controlling the disease while minimising toxicity.

## Objectives

### Primary

The primary objective is to compare the mean DAS(CRP)−28 over 12 weeks in adult patients with active RA commencing intramuscular or oral short-term bridging GC therapy who are planning on initiating, escalating or switching DMARD therapy.

### Secondary

The secondary objectives to be evaluated in adult patients with active RA commencing oral short-term bridging GC therapy who are initiating, escalating, or switching DMARD therapy are:

- To compare the effect of two intramuscular and two PO GC dose regimens on mean DAS(CRP)−28, American College of Rheumatology (ACR) response and European Alliance of Associations for Rheumatology (EULAR) modified Boolean remission over 24 weeks.
- To compare the effect of oral and intramuscular GC on the following:
  - Patient-reported outcomes (pain & arthritis activity, fatigue, quality of life, work productivity and impairment and functional status).
  - Analgesic use.
  - Toxicity.
  - Cumulative GC dose.
- To conduct an economic evaluation.
- To assess GC bridging therapy acceptability.
- To conduct a qualitative study of GC bridging therapy acceptability.
- To conduct two qualitative studies: one with patients who decide not to take part in the LEADER trial and one with healthcare professionals involved in delivering the LEADER trial at participating sites.

## Methods and analysis

### Trial design

**Figure 1:**
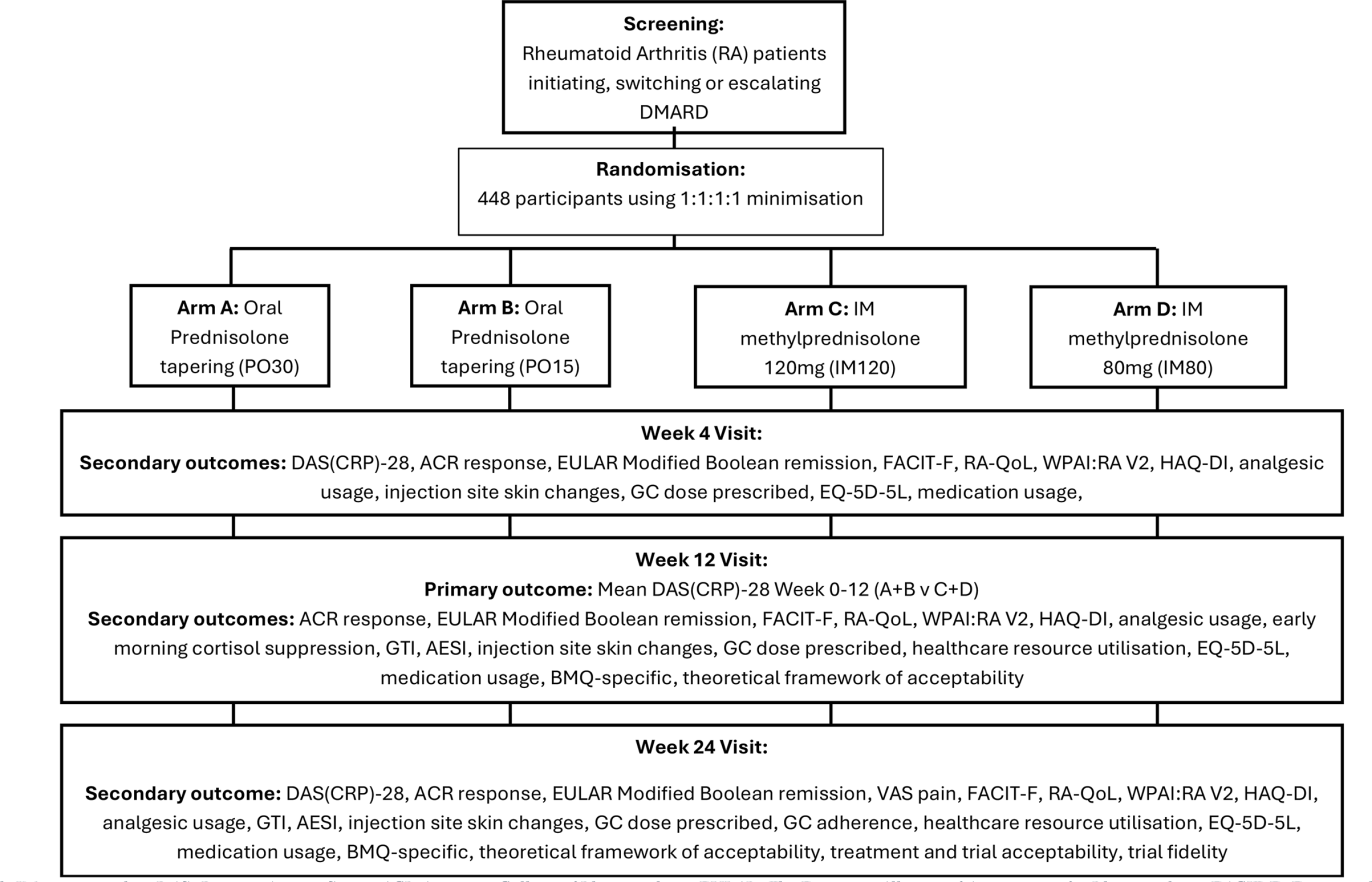
The LEADER trial flow chart. Weekly VAS pain scores and steroid diaries will also be collected. ACR, American College of Rheumatology; BMQ, Belief about Medicines Questionnaire; DAS, Disease Activity Score; EQ-5D-5L, EuroQol 5-Dimension 5-Level; EULAR, The European Alliance of Associations for Rheumatology; FACIT-F, Functional Assessment of Chronic Illness Therapy–Fatigue; GC, glucocorticoid; HAQ-DI, Health Assessment Questionnaire Disability Index; IM, intramuscular; PO, oral; QoL, Quality of Life; WPAI, Work Productivity and Activity Impairment.

LEADER is a multi-centre, randomised, open-label, four-arm, parallel-group clinical trial with an internal pilot phase, economic evaluation and qualitative sub-studies of acceptability (figure 1).

Participants will be randomised to one of the following arms (table 1).

**Table 1:** LEADER intervention arms

Arm	Treatment
Arm A (PO30)	Oral GC (prednisolone) tablets. Starting dose of 30 mg once daily tapered down each week over 5 weeks to 5 mg once daily. Total treatment duration is 6 weeks.
Arm B (PO15)	Oral GC (prednisolone) tablets. Starting dose of 15 mg once daily tapered down each week over 3 weeks to 5 mg once daily. Total treatment duration is 4 weeks.
Arm C (IM120)	One-off 120 mg intramuscular GC injection (methylprednisolone)
Arm D (IM80)	One-off 80 mg intramuscular GC injection (methylprednisolone)

GC, glucocorticoid.

The internal pilot phase will progress to the definitive trial if predefined progression criteria are reached. Stop-go criteria will be reviewed after 9 months of recruitment (table 2).

**Table 2:** Stop-go criteria for the internal pilot phase of the LEADER trial

Progression criteria	Red	Amber	Green
Number of sites opened	≤10	11–17	≥18
Total number of participants recruited	≤64	65–107	≥108
Recruitment rate/site/month	≤0.7	0.71–1.10	>1.10
Trial recruitment	<60%	60–99%	100%
Protocol deviation from the randomised route of administration on first prescription	>10%	6–10%	≤5%

### Participant recruitment

Participants are recruited from rheumatology outpatient clinics in the United Kingdom.

The following approaches are used to identify potentially eligible participants:

- Identification during rheumatology clinic visits/telephone appointments or contact via the advice line.
- Advertising of the trial in rheumatology outpatient clinics directing potential participants to the local site research team for further information.
- Advertising of the trial by The National Rheumatoid Arthritis Society (NRAS), a registered charity dedicated to improving the lives of adults diagnosed with RA and other organisations as a research opportunity on their social media outlets.

Potential participants who contact the advice line are sent the Participant Information Sheet (PIS; online supplemental file 2) and patient trial summary, which signposts the LEADER explainer animation.^18^ Materials are provided using any mode of communication routinely used by participating sites to contact patients.

The trial eligibility criteria are broad to reflect clinical practice in the UK. Eligibility can be confirmed by a medically qualified doctor or a nurse who routinely assesses rheumatology patients for GC bridging therapy and who has been delegated to do so by the Principal Investigator (PI), based on the below criteria and rationale:

### Inclusion criteria

- Aged 18 years or over—adult-only trial.
- A clinical diagnosis of RA—reflects clinical practice.
- Active disease defined as ≥3 tender and ≥3 swollen joints—reflects active disease.
- Planning on initiating/switching/escalating to one conventional synthetic (cs), biologic (b) or targeted synthetic (ts) DMARD—fulfils trial aims and allows retention of participants who may decline DMARD therapy when offered at medication counselling appointment.
- Willing and able to accept either trial arm allocation—fulfils trial aims.
- Willing and able to give informed consent—legal requirement and core principle of Good Clinical Practice.

### Exclusion criteria

- Oral, intramuscular or intra-articular glucocorticoid therapy within the past 28 days—efficacy, affects baseline disease control.
- Glucocorticoid therapy contraindicated (as determined by treating clinician)—safety.
- Diagnosed within the last 6 months with fibromyalgia or chronic widespread pain—efficacy, endpoint may be affected by active non-inflammatory disease.^19^
- Known to be pregnant or female patient trying to conceive—safety.
- Unstable or uncontrolled diabetes—safety.
- Participating or planning to participate in another interventional clinical study/trial during the trial period that, in the opinion of the investigator, could affect LEADER outcomes—improve quality of data collection and reduce missingness.
- Any other severe concomitant disease that, in the opinion of the investigator, might interfere with trial procedures and/or assessments—safety.

LEADER will be conducted in ~30 sites delivering NHS care with an anticipated 18-month recruitment period. The trial is supported by Trial Steering and Data and Safety Monitoring Committees in accordance with NIHR guidelines.^20^ Recruitment commenced on 4 July 2025, with an anticipated 16-month recruitment period. Quality assurance (QA) will be provided by the Oxford Clinical Trials Research Unit (OCTRU) QA team who will monitor this trial by conducting audits of the Trial Master File. Furthermore, the processes of receiving consent, registration, randomisation, provision of information and provision of treatment will be monitored by the central CTU trial team. Additionally, the trial may be monitored or audited by the sponsor or their authorised delegate, or at individual host sites in accordance with the current approved protocol, GCP, relevant regulations and standard operating procedures. The sponsor is responsible for publication approval.

### Randomisation

Randomisation will be performed following informed consent via an automated, secure web-based randomisation REDCap platform. Allocation will be minimised with a 1:1:1:1 allocation ratio to ensure balance for recruiting centre, number of previous DMARDs (0–1 vs 2 or more) and b/tsDMARD versus csDMARD commencement. Participants will be viewed as compliant if they received the correct prescription as per their randomised allocation, reflecting the pragmatic nature of the study.

### Trial visits

Participants will be assessed at baseline (pre-GC intervention) and at 4, 12 and 24 weeks. Visit windows have been incorporated to allow an element of flexibility. Data collected are summarised in table 3.

**Table 3:** LEADER data collection schedule

Data collected by site research team				
	Weeks post-randomisation			
	Baseline	4	12	24
Medical history	X			
ACR/EULAR 2010 Rheumatoid Arthritis classification criteria^35^	X			
Components of DAS(CRP)−28, ACR response and EULAR Modified Boolean remission	X	X	X	X
Medication usage: GC prescribed Cumulative GC usage Additional steroid therapy Analgesic use DMARD usage	X	X	X	X
IMP adherence	X	X		
Glucocorticoid Toxicity Index^36^	X		X	X
Skin changes at Injection site		X	X	X
AEs of special interest*			X	X
Healthcare resource utilisation	X		X	X
Data completed directly by participant				
Demographics	X			
Pain & arthritis activity VAS	Collected weekly			
Steroid diary (oral arm only)	Completed weekly			
FACIT-F (Fatigue)	X	X	X	X
RA-QoL (RA-Quality of Life)	X	X	X	X
WPAI:RA V2 (Work Productivity and Impairment)	X	X	X	X
EQ-5D-5L	X	X	X	X
HAQ-DI (Functional status)	X	X	X	X
BMQ (Medication beliefs)	X		X	X
Theoretical framework of acceptability			X	X
Data collected by site research team from central laboratory				
Early morning cortisol suppression			X†	

* AESI include gastrointestinal ulceration or bleeding, tendon rupture, fracture, heart failure and venous thromboembolism.

† Available from week 12.

ACR, American College of Rheumatology; AE, adverse event; BMQ, Beliefs about Medicines Questionnaire; DAS, Disease Activity Score; DMARD, disease-modifying anti-rheumatic drug; EULAR, The European Alliance of Associations for Rheumatology; GC, glucocorticoids; HAQ-DI, Health Assessment Questionnaire-Disability Index; IMP, Investigational Medicinal Product; VAS, Visual Analogue Scale.

Data will, wherever possible, be collected in electronic format with direct entry into a trial-specific REDCap database by site staff or participants.^21 22^ Trial data and associated metadata will be stored electronically in a suitable format in a secure server area maintained and backed up to the required standard. Access will be restricted to the responsible archivist and will be controlled by a formal access request.

### Blinding

To avoid observer bias, where possible, disease activity will be assessed using the DAS(CRP)−28 by a member of the research or usual care team blinded to the participant’s treatment allocation. Site staff will record in the REDCap database whether DAS(CRP)−28 was assessed blinded/unblinded. A sensitivity analysis will be undertaken to explore the impact blinding has had on the main analysis.

### Flare management

Based on the results of the CareRA trial, we expect that a number of LEADER participants will have active disease, defined as (DAS28-CRP)>3.2, from weeks 8 to 24 in Arm A (PO 30 mg prednisolone reducing in dose over 6 weeks)^23^ which may require additional bridging GC therapy. If additional GC therapy is medically indicated, it should be given within the treatment arm that the participant has received at baseline. This additional dose of GC therapy will be recommended by the local treating clinical team, as medically indicated. The cumulative dose of GC therapy received will be calculated. Protocol adherence of post-GC bridging therapy flare management will be evaluated during the internal pilot phase.

### GC toxicity and adverse event recording

GC toxicity will be measured using The Glucocorticoid Toxicity Index (GTI), a clinical outcome assessment developed for use in prospective randomised controlled trials to measure change in GC toxicity between two points in time and measures cumulative worsening and improvement of GC toxicity.^24^ GCs may cause secondary adrenal insufficiency (SAI) resulting in symptoms of fatigue, weakness and weight loss. A systematic review of SAI concluded that there is no administration form, dosing, treatment duration or underlying disease for which SAI can be excluded with certainty'.^25^ Lower morning cortisol levels have been demonstrated 12 weeks following intramuscular GCs^26^ but it remains unknown which GC dose and method of administration has a lower SAI risk. The LEADER trial will measure SAI once from week 12 as a key secondary outcome measure. Early morning cortisol level will be measured using an at-home self-collection blood sample so that participants do not have to visit a healthcare environment. Participants with conditions known to affect the hypothalamic–pituitary–adrenal (HPA) axis will be excluded. Should an abnormal cortisol result be identified, sites are advised to follow the steps set out in the National Institute for Health and Care Excellence (NICE) guideline NG243 Adrenal insufficiency: identification and management.^27^ The following are Adverse Events of special interest (AESI) in the LEADER trial:

- Gastrointestinal ulceration or bleeding.
- Tendon rupture.
- Fracture.
- Heart failure.
- Venous thromboembolism.

### Withdrawal procedures

Participants may fully or partially withdraw from the trial. Participants may also be withdrawn from the trial (or aspects of the trial) by their clinician if they believe the participant needs to be withdrawn. Where a participant expresses a wish to withdraw from certain aspects of the trial only, the site research team will determine which aspect(s) of the trial the participant wishes to withdraw from.

The aspects of the trial that the participant may request to withdraw from that will not constitute a complete withdrawal are as follows:

- No longer willing to complete trial questionnaires.
- No longer willing to complete weekly pain scores.
- No longer willing to take part in qualitative aspects of trial.
- No longer willing to provide at-home cortisol blood test.

If a participant experiences a toxicity, which in the view of the clinical team requires dose reduction, interruption or permanent discontinuation, this will be permissible without withdrawing the participant from the trial. Participants who lose capacity during the trial will be withdrawn. Data and samples collected up to the point of withdrawal will be retained and/or analysed.

### Qualitative sub-studies

Qualitative sub-studies will be conducted to explore perceptions and experiences of GC therapy, assess the acceptability of the trial and inform the development of a process evaluation of the intervention.

Qualitative studies will be undertaken with the following populations:

- Patients that decline participation in the LEADER trial—to explore the prospective acceptability of the trial and reasons for non-participation.
- LEADER trial participants—to explore the acceptability and experiences of GC bridging therapy within the trial context.
- LEADER healthcare professionals at LEADER trial sites—to explore perceptions of trial acceptability and fidelity, including views on intervention delivery, recruitment, and retention.

Interviews and focus groups will be used to explore the acceptability of the LEADER intervention, such as views about injections versus tablet GC bridging therapy, and trial processes, for example, contextual factors influential in participation and retention in the trial. Data collection and analysis will be informed by constructs from the Theoretical Framework of Acceptability^28^ and the Theoretical Domains Framework.^29^

### Sample size and justification

A sample size of 448 participants in the trial is needed. This is based on a target mean difference of 0.6 in the mean DAS(CRP)−28 over the 0–12 week period assuming SD of 1.3,^12^ two-sided statistical significance level of 5% and allowing for 10% missing data. The principal comparison (oral (PO) vs intramuscular) will have 99% power, and the two secondary comparisons of oral (PO30 vs PO15) and intramuscular (IM120 vs IM80) interventions, 90% power. This is a conservative calculation, given a linear mixed model will be used to estimate the estimand of interest adjusting for the randomisation variables and using all available DAS(CRP)−28 data. The target difference of 0.6 in the DAS(CRP)−28 is the magnitude of change generally considered to indicate an improvement in the disease condition.^30^

### Statistical analysis

Statistical aspects of the LEADER trial are summarised here with details fully described in a statistical analysis plan and health economic analysis plan that has been drafted and will be finalised prior to the final analysis data lock. No interim statistical analysis is planned.

#### Primary outcome

All pre-specified analyses will be conducted once all participant recruitment and follow-up is complete at the end of trial follow-up. The primary comparison will be according to randomised groups (oral vs intramuscular GC treatment, ie, groups A+B (PO30+PO15) vs groups C+D (IM120+IM80)) irrespective of non-compliance and treatment adherence, according to an intention-to-treat (treatment policy) approach. The main analysis of the primary outcome will use a linear mixed model with adjustment for the minimisation variables (site, number of previous DMARDS (0–1 vs 2 or more), type of DMARD: b/tsDMARDs vs csDMARDS), baseline DAS(CRP)−28 and time point (as a treatment by timepoint interaction term) as fixed effects and will compare the mean DAS(CRP)−28 between oral and intramuscular over 0–12 weeks. This reflects the estimand of principal interest for the outcome over the short term as a treatment policy. The model is anticipated to use an unstructured covariance matrix and maximum likelihood estimation. This approach assumes that missing data is missing at random (MAR) conditional on the other data collected. The DAS(CRP)−28 will also be compared at 24 weeks using the same linear mixed model.

The two secondary comparisons (oral groups A (PO30) vs B (PO15) and intramuscular groups C (IM120) vs D (IM80)) will also be carried out by conducting corresponding analyses on the respective randomised sub-samples which will be used to compare the oral and intramuscular intervention groups respectively according to allocation.

#### Secondary outcomes

Secondary outcomes will be analysed according to comparisons of interest as per the primary outcome using generalised linear regression/mixed models with adjustment for minimisation and baseline variables as appropriate.

#### Analysis principles

The principal analysis will be performed on the as-randomised analysis set, analysing participants with available outcome data in their randomised groups, irrespective of non-compliance and treatment adherence.

All treatment comparisons will be reported with 95% CIs, and a significance level of 5% will be used to test statistical significance.

#### Subgroup analysis

Exploratory subgroup analyses of DAS(CRP)−28 over weeks 0–12 will explore the possible treatment effect modification of Rheumatoid Factor/Anti-Citrullinated Protein Antibodies status and DMARD commencement (csDMARD vs ts/bDMARD) at baseline, by use of a treatment by subgroup factor interaction, and will be viewed as exploratory in nature.

#### Economic analysis

An economic evaluation will be undertaken to compare the cost-effectiveness of oral with intramuscular GCs from the English National Health Service (NHS)/Personal Social Services perspective. Two analyses will be performed. The primary analysis is a decision-analytic model-based cost-effectiveness analysis, with a lifetime horizon, extrapolating from the primary trial outcome. This approach will follow the NICE reference case to ensure the evidence will inform NICE RA clinical guidelines^7^ and so is directly applicable in a decision-making context in the English NHS. A within-trial economic analysis will also be conducted, using the prospective EQ-5D-5L and resource use data generated within the trial. The economic evaluations will be reported according to the Consolidated Health Economic Evaluation Reporting Standards (CHEERS 2022) statement.^31^ The decision-analytic model will be validated using the AdViSHE tool.^32^

#### Within-trial economic analysis

The within-trial economic analysis will use data collected prospectively during the trial. The analysis will compare the difference in total cost and total health outcomes for oral GCs and intramuscular GCs over the trial follow-up period (24-week time horizon). All participants randomised as part of the trial will be included (intention-to-treat analysis). As a pragmatic trial, the base case analysis will assume that the duration of GC treatment will follow the observed treatment patterns in each arm. healthcare resource utilisation per participant will be measured using a bespoke questionnaire.^33^ Individual-level costs will be created for each participant by combining the measured resource use with published unit costs from publicly available sources. Health outcomes will be expressed as quality-adjusted life years (QALYs). The EQ-5D-5L responses will be converted into health utility values using the method recommended by the NICE reference case at the time of data analysis. QALYs per participant will be calculated by the area under the curve method. Total cost and QALYs per arm will be estimated by generalised linear model regressions with multiple imputation to handle missing data and bootstrapping to handle uncertainty.

#### Model-based economic analysis

The primary analysis will be a probabilistic model-based economic analysis that compares the difference in total cost and total health outcomes for oral GC and intramuscular GCs over a lifetime time horizon (discount rate for cost and QALYs: 3.5%). This analysis will demonstrate the health economic impact of both GC regimens over an individual’s full lifetime pathway of care by extrapolating the primary outcome beyond the trial follow-up. Data sources to populate this model-based analysis will comprise primary data from the LEADER trial and relevant data from the published literature and expert opinion.

### Patient and public involvement

In 2023, in preparation for the funding application, two patient advisory group meetings were held to discuss the project proposal. The group comprised individuals from diverse backgrounds, including three Patient Research Partners (PRPs) and three public contributors, one of whom is a carer. Researchers and group members explored perceptions and experiences of steroid use, with members drawing on their personal knowledge of GC therapy, including its benefits for pain relief and short and long-term side effects. Group members expressed the view that injections may be perceived as ‘stronger’ than tablets and agreed on the need to explore this in a qualitative study.

Advisory group members raised concerns about the potential for patients with RA to experience a disease flare during the trial. They emphasised the need for flexibility to manage flares and cautioned against an overly prescriptive approach. In response, the LEADER trial will allow participants who flare to receive additional GC therapy. Cumulative GC dosages received will be recorded, and their impact examined. PRPs also stressed the importance of flexible scheduling for trial visits leading to the incorporation of visit windows within the trial design.

The group recommended creating a short video featuring a patient sharing their perspective on the importance of taking part in the trial and advised including multilingual subtitles to enhance accessibility. Working closely with our PRPs, we have co-designed a suite of materials to support recruitment and delivery of the LEADER trial. These include a recruitment poster, a one-page trial summary, data collection tools, patient information sheets, consent forms, the interview topic guides for the qualitative sub-studies and an explainer animation.^18^

### Ethics and dissemination

The LEADER trial received MHRA, HRA and Leicester Central Research Ethics Committee ethics approval as a Clinical Trial of Investigational Medicinal Product (CTIMP) using the combined review process in January 2025 (REC reference: 24/EM/0277, IRAS 1010280). Recruitment opened under Protocol Version 4.0. A substantial amendment (protocol version 5.0; 02 Dec 2025) was approved to remove visits at weeks 2 and 8 to reduce participant and site burden and reduce the exclusion criteria for previous GC therapy from 3 months to 28 days. The decision to reduce previous GC therapy from 3 months to 28 days was made following exploration of screening log data. This was deemed clinically acceptable as the effect of intramuscular GC therapy may be lower than 3 months.^34 35^ Participants will provide written informed consent (online supplemental file 3). All substantial protocol modifications would be approved by the MHRA/REC/HRA as required and communicated to sites and other relevant parties including trial registries.

The trial results will be published in an open-access journal and reported following the CONSORT guideline including any applicable extensions to this. The Template for Intervention Description and Replication (TIDieR) statement will be used for reporting the intervention. Where possible, plain English summaries will be published alongside the full paper, along with links to other digital media on the trial website to explain the trial result in an accessible format—ie, an explainer video and infographic. The statistical and health economics analysis plans will be published in open-access peer-reviewed journals before analysis is commenced. The results of this trial will be disseminated to the clinical community via presentations at national and international meetings. Traditional conference dissemination will focus on presentations to include the key professional stakeholders. Authorship of any publications arising from the trial will be determined in accordance with the International Committee of Medical Journal Editors (ICMJE) guidelines and any contributors acknowledged accordingly.

To ensure a broad campaign for public dissemination, we will target a range of social media outlets. We will seek to engage the NHS Dissemination Centre and plan to publish a ‘digital story’ as part of the ‘NIHR Signal’. In collaboration with our PRPs and NRAS, we will disseminate the results of the LEADER trial through journals, conferences, social media, newsletters and online workshops for patients, stakeholders and clinicians. A summary of the trial results for trial participants will be co-designed with clinicians and PRPs and distributed accordingly. The PIS includes a link to the trial website where participants will be informed that the study trial will be published. Newsletters and social media outlets will also be used to ensure the results of the trial are communicated to the wider community once they are available. On completion of the trial, we may deposit a fully anonymised dataset in an open data repository. Anonymised research data may also be shared with other organisations on request to the Chief Investigator and in accordance with the data sharing policies of OCTRU, the sponsor and funders.

## Discussion

The LEADER trial is designed to address a significant knowledge gap by identifying the most effective and safest way of using GCs in patients with uncontrolled RA who are starting an anti-rheumatic medicine. The primary analysis will assess whether an intramuscular GC injection or oral prednisolone therapy results in reduced disease activity over 12 weeks in patients commencing a DMARD therapy. Secondary analysis will provide evidence for GC dosage for a given administration regimen and patient acceptability. LEADER has broad eligibility criteria so that the findings will be externally valid. Additional GC therapy for a flare is permissible within the allocated treatment arm. LEADER will compare the effect of oral and intramuscular GC on cumulative GC dose received in the population recruited. We will monitor compliance with the administration regimen allocation.

A potential limitation is that the primary outcome measure is blinded where possible. Pragmatically we opted for blinding the research or usual care team where possible due to the number of participants we needed to recruit and concern that smaller sites may not have the capacity to deliver LEADER with the outcome measure fully blinded. A sensitivity analysis will be undertaken to explore the impact blinding has had on the main analysis.

Current guidelines offer no advice on dose, route of administration and duration of GC therapy in patients living with RA who are commencing a DMARD. LEADER will provide evidence for the most effective GC regimen, while limiting toxicity and being acceptable to patients, in this population.

## Supplementary material

10.1136/bmjopen-2026-119885 online supplemental file 1

10.1136/bmjopen-2026-119885 online supplemental file 2

10.1136/bmjopen-2026-119885 online supplemental file 3
