# Supplementary material for: Clinical and cost-effectiveness of oral versus intramuscular glucocorticoids in rheumatoid arthritis: protocol for a multicentre randomised controlled trial with economic evaluation and qualitative sub-study (LEADER trial)
Source: BMJ Open. 2026 Jul 10;16(7):e119885. doi: 10.1136/bmjopen-2026-119885 (PMC13358284; doi:10.1136/bmjopen-2026-119885)
Supplement: online supplemental file 1 [file bmjopen-16-7-s001.docx]

| **Site name** | **PI** |
| --- | --- |
| **Airdale NHS Foundation Trust** | Dr Hanu Reddy |
| **Birmingham & West Sandwell NHS Foundation Trust** | Dr Paola de Pablo |
| **Buckinghamshire (Stoke Mandeville) NHS Trust** | Dr Malgorzata Magliano |
| **Darlington Memorial Hospital (Country Durham NHS Foundation Trust)** | Dr Sanjay Pathare |
| **East Sussex Healthcare NHS Trust** | Dr Sam Panthakalam |
| **Guy's & St Thomas' NHS Foundation Trust** | Dr Charles Raine |
| **Homerton Healthcare NHS Foundation Trust** | Dr Claire Gorman |
| **King's College Hospital (King's College London NHS Foundation Trust)** | Dr James Galloway |
| **Manchester University NHS Foundation Trust** | Professor Anne Barton |
| **Mersey & West Lancashire NHS Trust** | Dr Julie Dawson |
| **Mid Cheshire Hospitals NHS Trust** | Dr Kiran Putchakayala |
| **Mile End (Barts QMUL Health NHS Trust)** | Dr Felice Rivellese |
| **Northampton General Hospital** | Dr James Taylor |
| **Northern Care Alliance - Fairfield General** | Dr Sophia Naz |
| **Northern Care Alliance - Rochdale Infirmary** | Dr Dhivya Ghandi Das |
| **Pennine MSK Partnership** | Dr James Bluett |
| **Plymouth University Hospital NHS Trust** | Dr Owen Moore |
| **Princess Alexandra Hospital NHS Trust** | Dr Khalid Ahmed |
| **Royal Wolverhampton NHS Trust** | Dr Sabrina Raizada |
| **Sussex University Hospital** | Dr Cristina Tacu |
| **University College London Hospital** | Dr Madhura Castelino |
| **West Suffolk NHS Foundation Trust** | Dr Shweta Bhagat |

**LEADER Sites and Principal Investigators**
