# Supplementary material for: Clinical and cost-effectiveness of oral versus intramuscular glucocorticoids in rheumatoid arthritis: protocol for a multicentre randomised controlled trial with economic evaluation and qualitative sub-study (LEADER trial)
Source: BMJ Open. 2026 Jul 10;16(7):e119885. doi: 10.1136/bmjopen-2026-119885 (PMC13358284; doi:10.1136/bmjopen-2026-119885)
Supplement: online supplemental file 2 [file bmjopen-16-7-s002.pdf]

To be printed on Trust/equivalent headed paper

ORAL VERSUS INTRAMUSCULAR GLUCOCORTICOIDS IN RHEUMATOID ARTHRITIS (LEADER)

Participant Information Sheet (PIS)

You are being invited to take part in a research study (also known as a clinical trial) called the LEADER study. The LEADER study is trying to find out the best way of using steroids which are routinely used to treat the symptoms of rheumatoid arthritis (RA).

Doctors are wanting to find out what is the best way for individuals with rheumatoid arthritis to receive these steroids – either as a one-off injection into a muscle, which is called “intra-muscular”, or as tablets which need to be swallowed daily for either 4 or 6 weeks to see which is better for controlling the symptoms of rheumatoid arthritis. The study will also compare different doses (amounts) of the steroids.

You are being invited to take part as your rheumatology care team is recommending you take steroids to treat the symptoms of your rheumatoid arthritis. Before you decide whether to take part, it is important for you to understand why the research is being conducted and what it will involve. Please take time to read the following information carefully before deciding whether to take part and discuss it with others if you wish. You may also like to watch a short video about the study which you can access using this QR code.

QR placeholder

It is important that you have enough time to consider if you wish to take part in the LEADER study. You may also wish to discuss taking part with your family/friends. If you prefer, you can take this information away with you and return on a separate agreed clinic visit to take part in the LEADER study. If you wish to do this, then you will receive your steroid treatment at your next visit.

Please ask if there is anything that is not clear or if you would like more information.

Thank you for taking the time to read this.

About the study

➢ **Who will conduct the study?**

This study is a collaboration between doctors, nurses and researchers who are based at The University of Manchester, Manchester University NHS Foundation Trust, King's College Hospital NHS Foundation Trust, University College London Hospitals NHS Foundation Trust, The Queen's University of Belfast and the University of Oxford.

The person who has overall responsibility for the study is Dr James Bluett who is an honorary consultant rheumatologist and researcher at the University of Manchester. The University of Manchester is sponsoring the study. This means that it is legally responsible for the organisation of the study.

The study is being organised by the Oxford Clinical Trials Research Unit (OCTRU) at the University of Oxford (referred to as the trial office).

➢ **What is the purpose of the study?**

Rheumatoid arthritis (RA) causes joint pain and swelling which affects people's day to day activities and quality of life. Treatment for rheumatoid arthritis usually involves medicines called disease-modifying anti-rheumatic drugs (DMARDs) such as methotrexate or biologic therapies. DMARDs help to stop rheumatoid arthritis from getting worse, however they can take time to fully work.

Whilst waiting for DMARDs to work, patients are offered treatment with steroids which act quickly to control the symptoms. These steroids can be given as an injection into the muscle ("intra-muscular") or as daily tablets which need to be swallowed. Although steroids are good at controlling symptoms of rheumatoid arthritis, they should not be taken long term, and they can cause side-effects.

Doctors currently do not know whether it is better to give steroids for rheumatoid arthritis as an intra-muscular injection or as tablets and what dose (amount) of these steroids is best to use. The LEADER study is comparing different doses of steroids which may be given either as a one-off injection or tablets taken daily for either 4 or 6 weeks to see which is better at controlling the disease.

LEADER is a randomised study, which means that the way and amount of steroid treatment a patient receives is randomly chosen by a computer. Those who agree to take part will be given at random one of the following four steroid treatments:

- Higher dose steroid tablets
- Lower dose steroid tablets
- Higher dose steroid injection
- Lower dose steroid injection

All of these treatments are currently used to treat rheumatoid arthritis but we want to know which works best which is why we are doing the LEADER study.

➢ Am I suitable to take part?

We are inviting people aged 18 years or over who have active rheumatoid arthritis and who are about to start treatment with a DMARD medicine, or changing their current DMARD medicine, to consider taking part in the LEADER study.

You will not be able to take part if you have uncontrolled diabetes, are pregnant, have received steroid treatment within the past 28 days or have been diagnosed with fibromyalgia or chronic widespread pain in the last six months.

The study aims to have approximately 448 individuals taking part from across the UK.

➢ Will the outcomes of the study be published?

Yes. The results of the LEADER study will be published. The results will be shared with you, as well as healthcare researchers and professionals to improve future patient care. You will not be identified in any results. The results will also be made available on the study website here: https://leader.octru.ox.ac.uk.

➢ Who has reviewed the research project?

All research in the NHS is looked at by an independent group of people, called a Research Ethics Committee, to protect participants' interests. This study has been reviewed and given favourable opinion by Leicester Central Research Ethics Committee.

➢ Who is funding the research project?

The study is funded by the National Institute for Health and Care Research (NIHR) through a research grant.

What would my involvement be?

➢ What would I be asked to do if I took part?

A summary of what taking part in the LEADER study would involve is provided below with further, more detailed information, later in this section. If you agree to join the study, you will be part of the study for about 24 weeks (6 months).

If you agree to take part in the LEADER study, in addition to the clinic visits that you would have anyway as part of your usual care we would ask you to:

- Answer some questionnaires.
- Rate your pain once a week for the duration of the study (24 weeks).
- Agree to your hospital or clinic appointments being longer.
- Give some blood for the study.
- Do a simple finger-prick blood test at home.

- If you are in either of the groups taking steroid tablets, complete a paper diary to record taking your tablets.

This diagram shows when there would be anything asked of you for the LEADER Study.

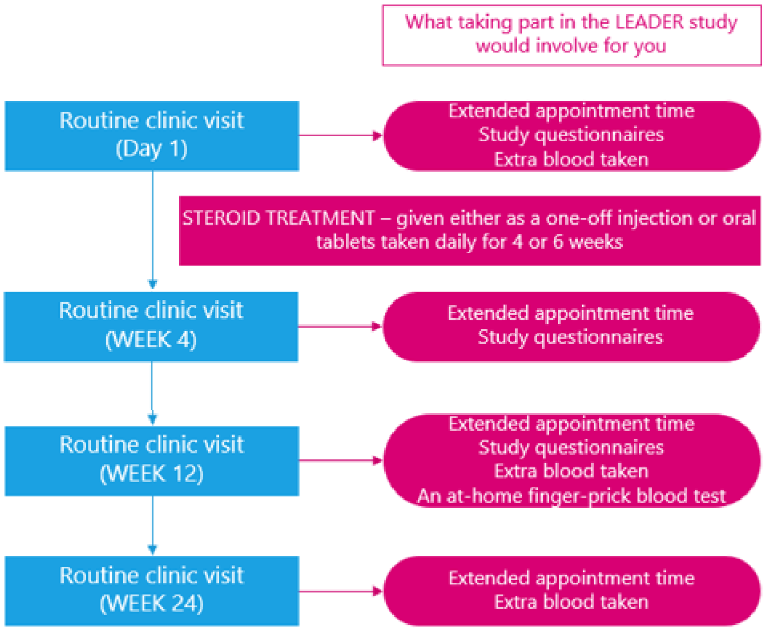

Current national guidelines suggest that patients with active RA are seen in clinic monthly. If you decide to take part in the LEADER study, your routine rheumatology clinic appointments will take longer than usual to give you time to complete the additional study assessments (approximately 45 minutes longer). If convenient, you can also complete the questionnaires at home before your clinic appointments to help save time (except for your first clinic visit).

➢ Day 1 of your taking part in the study

You will be asked to sign a consent form to confirm that you have read all the information in this information sheet and understand fully what taking part involves. A member of the study team at your clinic/hospital will check your medical history to check it is safe for you to take part.

You will be asked some additional questions about your health for the purpose of the study and we will collect some information from your medical records about your medical history, rheumatoid arthritis, and treatments you have had previously. You will also be asked to complete some study questionnaires about your rheumatoid arthritis, quality of life and how your rheumatoid arthritis affects you.

During your clinic appointment your rheumatology care team will examine you to check your rheumatoid arthritis signs/symptoms and for any treatment side effects. Your rheumatology care team will also perform some other routine health checks such as checking your height, weight and blood pressure.

As part of your usual rheumatoid arthritis care, you will normally have a blood test which is used by your rheumatology care team to check how active your rheumatoid arthritis is. We would like to do some additional tests at the same time for the LEADER study. This will allow us to look in more detail at how your body reacts to the steroids. The additional tests will be carried out by your clinic/hospital's usual laboratory and the results reported back to your rheumatology care team in the usual way. Your care team will check the result of these tests and if they are concerned in any way they will contact you to discuss the result with you and what this means. They may also inform your GP. For most people who join the LEADER study the additional blood tests will not require any additional blood to be taken, but this depends on which DMARD you are starting and can vary between clinics so it is possible that up to 15ml (3 teaspoons) of extra blood will need to be taken for the study. Your rheumatology care team will be able to tell you if extra blood will need to be taken for the study.

You will then be randomised (assigned) to one of four different treatment groups (also called arms) of the study. These are shown in the diagram below.

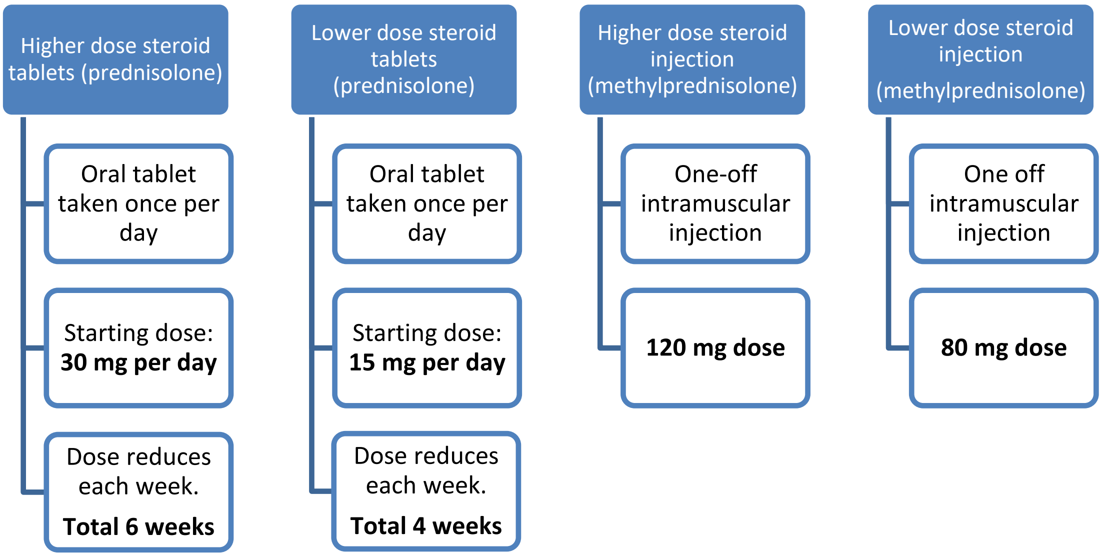

Your rheumatology care team will tell you which treatment you have been randomised to receive. We will also inform your GP that you are taking part in the study and which treatment you have been randomised to.

In two of the groups you would get a one-off injection of a steroid drug called methylprednisolone. The difference between the two groups is the amount of steroid you would get (120 mg or 80 mg). If you are randomised to receive a steroid injection, you will be given a single injection of the steroid into your muscle during your clinic appointment.

In the other two groups you would be asked to take steroid tablets called prednisolone once a day, at the same time each day for either 6 or 4 weeks. If you are put in the 6-week group the starting dose will be

30 mg a day. In the 4-week group the starting dose will be 15 mg a day. In both of these groups the amount of steroid you take decreases each week - this is shown in the table below.

**Week**	**Higher dose steroid tablets**	**Lower dose steroid tablets**
Week 1:	30 mg daily	15 mg daily
Week 2:	20 mg daily	10 mg daily
Week 3:	12.5 mg daily	7.5 mg daily
Week 4:	10 mg daily	5 mg daily
Week 5:	7.5 mg daily	END OF TREATMENT
Week 6:	5 mg daily	

Prednisolone tablets come in different strengths including 2.5mg, 5mg and 10mg tablets. This means you will often need to take more than 1 tablet a day to take the required dose, especially for the first few weeks of your treatment. You will receive a prescription for the tablets and you will need to get the tablets from your hospital or clinic's local pharmacy after your clinic appointment. The pharmacy that provides your tablets will give you the correct number of tablets, with instructions on the how many to take each day. All the tablets they give you may be the same strength such as the 2.5 mg tablets or they may give you different strengths to take on different weeks. Whichever they do, you will get clear instructions on what to take on the boxes of tablets or in a document called a treatment plan. We will also give you a study diary to record the steroid tablets you take whilst taking part in the study. You will be asked to bring your study diary with you to your clinic visits.

If you are put in one of the groups that will receive tablets and pay prescription charges we will reimburse the cost of the prescription.

You will then need to attend follow-up appointments at 1 month (Week 4), 3 months (Week 12) and 6 months (Week 24) to monitor your rheumatoid arthritis and any side effects of the steroid treatment in the same way that you would be if you were not taking part in the study. These appointments will take a little longer than usual. Further information about what will happen at each of these visits is given below.

- **Weekly Pain Rating**

Every week you are in the study you will be asked to answer one question to tell us how good or bad your pain is. We can send you a text message or email each week with a link to the question. This should take no longer than 2 minutes to complete each week. If you forget to answer this question we will send you a reminder 48 hours later.

Alternatively, we can provide you with a paper diary to record your pain scores in. You would need bring this to your clinic appointments.

- **Follow-up visits (approx. Week 4 and Week 12 of the study)**

You will need to attend further follow-up visits with your rheumatology care team to monitor your rheumatoid arthritis. You would normally have clinic visits at these timepoints even if you choose not to join the study but if you do take part in the LEADER study these visits will be about 30 minutes longer. When you come back to clinic for these routine follow-up visits, your rheumatology care team will once again do some further checks to monitor your rheumatoid arthritis and measure any steroid side effects. This will include a blood test which will be sent to your clinic/hospital's usual laboratory.

At your Week 12 visit, we will do some additional tests on your blood for the purpose of the LEADER study to look into potential side effects of treatment. Like at the Day 1 visit, for most people, the additional blood tests will not require any additional blood to be taken, but this can vary between clinics and depending what DMARD you are starting so it is possible that up to 15ml (3 teaspoons) of extra blood will need to be taken for the study. Your rheumatology care team will also perform some other routine health checks such as checking your weight and blood pressure.

Again at these visits, you will be asked some additional questions about your health for the purpose of the study and we will collect some information from your medical records about your health. You will also be asked to complete some study questionnaires, or you can choose to complete these before your visits to save time.

- **Week 12 - Additional at-home finger prick blood test**

Taking steroids can stop your adrenal glands from making enough of a hormone called cortisol and cause something called adrenal insufficiency but this is not usually checked as part of routine care. As part of the LEADER study we would like to look at the effect of giving steroids on the production of cortisol. To do this, you will be asked to provide a small blood sample using an at-home finger-prick test kit shortly after your Week 12 clinic visit. The blood sample needs to be taken between 8am-9am, it is therefore an at-home sample so that you do not have to travel to hospital early in the morning to have it done. A kit which contains everything you need to take the test along with instructions will be provided. The research team at your clinic/hospital will ask you where you wish the testing kit to be sent and you will receive this by post. You will be asked to take this finger-prick test between 8am-9am on a Monday, Tuesday, Wednesday or Thursday and return this by post (postage will be pre-paid) the day the test is taken.

Your doctor will check the result of your test and if they are concerned in any way, they will contact you to discuss the result with you and what this means. Your doctor may also contact your GP.

If you have an existing condition which is known to affect cortisol production or you are a shift-worker working nights (which also affects the body's cortisol production) then you will not be asked to do this. A member of the study team will discuss this with you and let you know if this applies to you.

The testing of your at-home finger prick blood test will be carried out by a company called Forth® who will also provide the test kit. Details of the information that will be provided to Forth to enable them to send the test kit to you and analyse the sample, can be found later in this information sheet in the section called Data Protection and Confidentiality.

- **Week 24 (6 Months)**

You will need to attend a further follow-up visit, as you would do if you were not part of the study, with your rheumatology care team to monitor your rheumatoid arthritis and the effects of the steroid treatment. If you take part in the LEADER study this visit will be about 30 minutes longer. During this visit your rheumatology care team will examine you to check your rheumatoid arthritis signs/symptoms. This will include a blood test which will be sent to the clinic/hospital's usual laboratory. Like at the Day 1 visit, for most people, the additional blood tests will not require any additional blood to be taken but this can vary between clinics and depending what DMARD you are starting, so it is possible that up to 15ml (3 teaspoons) of extra blood will need to be taken for the study. Your rheumatology care team will also perform some other routine health checks such as checking your weight and blood pressure.

In the same way as during your other visits, you will be asked some additional questions about your health for the purpose of the study and we will collect some information from your medical records about your health. You will also be asked to complete some study questionnaires, or you can choose to complete these before your visit to save time.

Your participation in the study will end after this visit. Once your study involvement has ended, you will continue to receive your usual care from your rheumatology care team. You will not receive any further steroid treatment through the LEADER study and any further steroid treatment that you need will be determined by your rheumatology care team and not the LEADER study team.

- **FAQs about the study**

What will the blood samples be used for?

Blood samples collected for the purpose of the study will be used to test the level of markers in your blood that measure how active your rheumatoid arthritis is and any side effects from steroid treatment, and the hormone cortisol. The finger prick blood test for measuring your cortisol will be sent to Forth®, the company who are carrying out these tests for us, and your sample will be analysed within 2 working days of receipt. All other blood samples will be processed by your NHS hospital site according to their local policies. The blood samples and finger prick blood sample will not be used for any genetic testing and will be securely stored until the sample is destroyed after testing.

What will the questionnaires ask about?

The questionnaires used in this study are routinely used in research studies in the UK. They will ask about how you feel about your rheumatoid arthritis, your health and how you are able to do things. No questions are compulsory to answer.

How will I be able to complete the study questionnaires?

It will take between 15 and 30 minutes to complete the study questionnaires.

To reduce the length of your clinic visits, you have the option to complete the study questionnaires before your clinic visits. We can send you an email or text message to let you know when these need completing with a link to an electronic questionnaire to complete, if you would be happy with this. You can decide if you prefer an email or text message reminder.

If you prefer you can choose to complete the questionnaires at your clinic visit.

We would also like your permission to contact you by phone or email if there is a problem with your questionnaire, such as they are not all answered.

Whichever method you choose, your study team will be able to check that the questionnaires have been completed and see the answers you have provided.

- **Optional parts of the study**

If you decide to take part in the LEADER study there are 2 optional parts we would like you to consider taking part in. You can choose to take part in both, one, or none of these optional parts, but still take part in the LEADER study.

1. <u>Optional interview so we can better understand your experience of the LEADER study</u>

So we can understand your experience of taking part in the LEADER study and how you found the steroid treatment, we would like to talk to people who take part in the study in an individual or group interview. So that we collect the experiences of a range of people, we will invite between 15-20 of those who have agreed to take part in the study and agree to be interviewed. This means that even if you agree to be interviewed, we may not interview you. We would talk to you either individually at a one-to-one telephone interview or as part of an online group interview also known as a focus group of 3-6 LEADER study participants – you choose which of these you would be happy to do, if at all. If English is not your first language, or you have a hearing impairment, you are welcome to have a family member or friend join the telephone interview to assist you.
If you are selected to be interviewed, your contact details will be securely provided to the member of the LEADER interview research team so they can contact you by telephone or email to explain the process and arrange a mutually convenient time for the interview. The LEADER researchers conducting these interviews are researchers based at King's College Hospital NHS Foundation Trust and the University of Manchester. With your consent we will record the audio of these interviews. However, we want you to feel comfortable with the recording process at all times and the researcher will check that you are happy before they start recording the interview. You will be free to ask the researcher to stop recording at any time. The conversation will then be typed up (transcribed) by a member of the LEADER interview research team or a University of Manchester approved transcription service and anonymised so it can be analysed. Once typed up we will delete the recording. The recording will remain secured within the unit responsible for these interviews at The University of Manchester until it is irrevocably deleted. The interview will last approximately 50 minutes. If you take part in an interview or focus group we can also offer a payment of £20 to cover additional expenses.

If you agree to take part in an interview you are also agreeing to us publishing direct quotes of what you say in the interview, along with your age and sex. These will always be in an anonymous format so you could never be identified from them.

2. Optional permission to contact you in the future

We would like to be able to keep in touch with you for various reasons but to do this we need your permission. The consent form will ask you to let us know if you are happy for us to keep hold of your contact details securely for the following reasons:
1. So we can contact you in the future to find out about your health and rheumatoid arthritis treatment. If you agree to this, we will hold your contact details for up to 3 years following the end of the study.
2. So we can provide you with a summary of this study. Please be aware it can take as long as 12 months after the end of the study for us to be able to send you this summary. You can choose whether you would like to receive this summary by post or email.

Please remember that both of these are **optional** parts of the LEADER study and you can take part in the study without agreeing to either of them.

➢ What will happen if my condition flares up and I need further treatment?

If you experience a flare of your rheumatoid arthritis whilst taking part in the study your rheumatology care team will discuss the treatment options with you and you will receive additional treatment as directed by your care team. Taking part in the LEADER study will not stop you from receiving any further treatment you need. We will however ask that you are given the same type of treatment as you received in the study i.e. if you were randomised to steroid tablets, you will be given more steroid tablets and not an injection.

If you do receive any additional tablet steroid treatment we will ask you to record it in the study diary we give you.

➢ Are there any benefits of taking part, and what are the risks?

All of the treatments being used in the LEADER study are currently used to treat rheumatoid arthritis but we want to know which works best. Like all medicines, steroids can cause side effects, although not everybody gets side effects. Your doctor will discuss these with you.

Information about the side effects of steroids and steroid injections is available on the Versus Arthritis website. You can also access these via the QR codes:

Steroids: https://www.arthritis-uk.org/information-and-support/understanding-arthritis/arthritis-treatments/drugs/steroids/

Steroid injections: https://www.arthritis-uk.org/information-and-support/understanding-arthritis/arthritis-treatments/drugs/steroid-injections/

A printed copy of this information may also be provided with this information sheet. Please ask a member of the research team for this if you would like this.

A benefit of taking part in the study is that unlike in routine care your cortisol level will be checked to see if the steroids are affecting your adrenal glands. If they are, this test would mean this is picked up more quickly than in routine care.

➢ **Will I be compensated for taking part?**

You will not be paid for taking part in the study, however we can provide a payment of £30 for each of the 4 study visits. These payments are to cover any additional expenses and your time to attend the extended clinic visits. If £30 is not enough to cover your additional costs, we may be able to cover additional costs over £30 but we will need to agree to this in advance and need you to explain your costs and provide receipts.

If you have caring responsibilities we can reimburse you up to £25 for additional costs incurred to attend the Day 1 visit and up to £10 per visit for additional costs incurred to attend the Week 4, 12 and 24 visits.

If you are randomised to receive steroid tablets and you pay prescription charges we will reimburse you £10 to cover the prescription cost.

You can choose whether you receive these payments as a voucher (electronic voucher sent by email, or physical voucher given to you at the visit or sent by post) or directly into your bank account. We will not require any documents such as receipts to make the payments. Payments made directly into your bank account will require staff at the University of Oxford to have your bank details. The information needed by the University of Oxford to make this payment would be collected at your clinic visit and provided securely to the University of Oxford so the payment(s) can be made. The next section explains who will have access to this information and how long it will be stored for.

➢ **What happens if I do not want to take part or if I change my mind?**

It is up to you to decide whether or not to take part. If you would like to take part you should tell the person who told you about the study. If you do decide to take part you will be given this information sheet to keep and will be asked to sign a consent form. If you decide to take part you are still free to withdraw at any time without giving a reason and without any impact on yourself but the study team and trial office will keep any data collected up to the time you withdraw for the purposes of the study, including data collected from any blood samples you had already given. We will not collect any further data about you and you will not be sent a summary of the results of the study. If you decide not to take part you do not need to do anything further.

If you do not want to take part in the LEADER study, a member of your rheumatology care team will ask you if you would like to take part in a "decliner" interview or focus group to discuss your reasons for not

wanting to take part. This is because we would also like to know why patients decline to take part in the study. Whether you decide to take part in this interview or focus group or not is up to you and it will not affect your clinical care. If you take part in a “decliner” interview or focus group, we can also offer a payment of £20 to cover additional expenses. The interviews will take place either online or by telephone.

- **What if something goes wrong?**

If you have a concern about any aspect of this study, please speak with your hospital care team or the research team. They will do their best to answer your questions.

In the unlikely event that something does go wrong and you are harmed during the research you may have grounds for a legal action for compensation against the University of Manchester or your NHS Trust, but you may have to pay your legal costs. The normal National Health Service complaints mechanisms will still be available to you.

The University of Manchester as the trial Sponsor will arrange insurance for research involving human subjects that provides compensation for non-negligent harm to research subjects occasioned in circumstances that are under the control of the University of Manchester, subject to policy terms and conditions.

- **How have patients and the public been involved in this study?**

The LEADER study has been designed with patients and healthcare professionals. A group of patients have also reviewed this information sheet and patients are part of some of the groups that oversee the running of the study.

[continues on next page]

Data Protection and Confidentiality

This section of the information sheet explains how we would use and protect your data.

➢ What information will you collect about me?

In order to participate in this study we will need to collect information that could identify you, called "personal identifiable information". Specifically we will need to collect:

- Your name
- Email address – if you agree to us sending you information and questionnaires by email
- Postal address – so we can send you the finger prick blood test and, if you agree, information such as newsletters by post
- Mobile number - if you agree to us contacting you by SMS or call about the questionnaires and pain rating
- Date of birth
- Record of consent
- Information about your arthritis and its treatment and your medical history
- Recordings of your voice during the interview/focus group discussion, if you agree and are chosen to take part.

We also need to collect the following sensitive information:

- Sex (male/female)
- Ethnicity - optional
- Highest education level - optional

Individuals from <insert Trust/equivalent name> will access your medical records to collect information for the study. The central coordinating team based at the University of Oxford, and the research team and clinical care staff at your hospital/clinic will have access to this information.

➢ Under what legal basis are you collecting this information?

We are collecting and storing this personal identifiable information in accordance with UK data protection law which protect your rights. These state that we must have a legal basis (specific reason) for collecting your data. For this study, the specific reason is that it is "a public interest task" and "a process necessary for research purposes".

➢ What are my rights in relation to the information you will collect about me?

You have a number of rights under data protection law regarding your personal information. For example, you can request a copy of the information we hold about you, including audio recordings.

If you would like to know more about your different rights or the way we use your personal information to ensure we follow the law, please consult our Privacy Notice for Research (http://documents.manchester.ac.uk/display.aspx?DocID=37095).

Sometimes your rights may be limited if it would prevent or delay the research. In this study your rights are limited, as we will retain the information collected about you up to this point if you choose to withdraw.

- **Will my participation in the study be confidential and my personal identifiable information be protected?**

In accordance with data protection law, The University of Manchester and the University of Oxford are the Data Controllers for this project. This means that we are responsible for making sure your personal information is kept secure, confidential and used only in the way you have been told it will be used. All researchers are trained with this in mind, and your data will be looked after in the following way:

All information collected about you for the purpose of this study will be treated as confidential. The only exception to this is if, during the study, we have concerns about your safety or the safety of others, we will inform your GP or care team. We will be using information from you and your hospital records in order to undertake this study and will use the minimum personally-identifiable information possible. You will be given a unique study number (ID) number and all data and results will be stored using this.

All information collected about you will be stored at the University of Oxford in a secure password-protected database, accessible only by authorised members of the study team.

We will store any research documents with personal information, such as consent forms, securely at the University of Oxford and/or The University of Manchester for 10 years after the end of the study, as part of the research record. The link between your name and study number will be kept for 10 years after the end of the study as part of these records. With your permission, the University of Oxford will keep your contact details for a maximum of 12 months after the study has finished to be able to send you a summary of the results of the study. You will be able to choose if you want the summary sent by email or post, or not at all. If you give permission for us to contact you in the future, the University of Manchester will retain your name and contact details for 3 years after the end of the study.

A copy of the consent form from this study will be kept in your medical records for as long as those records are retained.

So that the finger prick blood test kit (cortisol test) can be provided and the blood sample you provide for this test can be analysed the following information will be provided to Forth®, the company who are carrying out these tests for us:

- Your name & postal address – so they can send you the kit.
- Date of birth & sex – to be able to register and identify you on their system.

Forth® will retain this information for as long as necessary for the analysis of the full study results.

At the end of the trial, Forth® will transfer applicable data to the University of Manchester and will securely archive their own data and associated meta-data for 10 years from the end of the LEADER study. After this point, it will be permanently and securely destroyed so that it cannot be retrieved.

To be reimbursed for any additional costs and the time you give to study via BACS payment, your name, postal address, email address and bank details will be shared with the University of Oxford's Finance team. These details will be securely retained by the Finance team for a period of up to 7 years for audit purposes only and then destroyed. It will not be used by them for any other purpose. If you opt to be provided with shopping vouchers then these will be given to you by the study team who are running the study.

The name and contact details of those being invited to interview will be provided by the University of Oxford in a secure way to the researchers at the University of Manchester who are carrying out this part of the study Manchester will store this information securely.

Your participation in an interview will be audio recorded in Microsoft Teams and your personal data will be processed by Microsoft. This may mean that your personal data is transferred to a country outside of the European Economic Area, some of which have not yet been determined by the United Kingdom to have an adequate level of data protection. Appropriate legal mechanisms to ensure these transfers are compliant with the Data Protection Act 2018 and the UK General Data Protection Regulation are in place. The recordings will be removed from the above third-party platform and stored on University of Manchester managed file storage as soon as possible following the completion of data collection.

Transcription will be undertaken by a University of Manchester approved supplier who are only allowed to process your personal data for specified purposes and in accordance with our instructions.

We may disclose your personal data to our third-party service providers to carry out activities specifically for the purpose of this research study and as explained in this information sheet for example text messaging service providers/companies to send study-related text messages to you. Any third-party service providers are required to take appropriate security measures to protect your personal data in line with University of Oxford policies. We do not allow our third-party service providers to use your personal data for their own purposes, but rather to only process your personal data for specified purposes and in accordance with our instructions.

Your rheumatology care team will use contact details held in your medical record to contact you as required about the research study e.g., to schedule clinic visits.

With your consent, anonymised information will be shared in order to support additional research in accordance with UK Policy Framework for Health and Social Care Research or for related teaching purposes. The information that will be shared will not identify you and will not be combined with other information in a way that could identify you. It will only be used for the stated research and teaching purposes and will not allow you to be contacted nor will it be used to affect your care. It will not be used to make decisions about future services available to you, such as insurance.

At the end of the study we may deposit a fully anonymised dataset in an open data repository such as Figshare at the University of Manchester Library. Researchers at other institutions and others can request access to the anonymised data directly from the repository or from the study team directly and use it for further research or to check our analysis and results. We will also provide an anonymised copy of the data collected using the Glucocorticoid Toxicity Index with the company who developed the index, as this index is used within the LEADER study.

Please also note that individuals from The University of Manchester, University of Oxford, your hospital Trust/clinic or regulatory authorities may need to look at the data collected for this study to make sure the project is being carried out as planned or in the event of an incident. This may involve looking at identifiable data. All individuals involved in auditing and monitoring the study will have strict duty of confidentiality to you as a research participant.

Students and trainee doctors based at The University of Manchester may also be given access to the data collected in this study so they can do additional analyses. These people would not know your identity.

If you would like more general information on how researchers use data about patients, please visit: www.hra.nhs.uk/information-about-patients/

What if I have a complaint?

- **Contact details for complaints**

If you have a complaint that you wish to direct to members of the research team, please contact a member of the research team at your hospital or your study doctor – their contact details can be found at the end of this information sheet or you can contact the LEADER study team at the OCTRU.

LEADER study team

Tel: 0808 281 5345

Email: leader@ndorms.ox.ac.uk

If you wish to make a formal complaint to someone independent of the research team or if you are not satisfied with the response you have gained from the researchers in the first instance, then please contact:

The Research Ethics Manager, Research Office, Christie Building, The University of Manchester, Oxford Road, Manchester, M13 9PL, by emailing: research.complaints@manchester.ac.uk or by telephoning 0161 306 8089.

If you wish to contact us about your data protection rights, please email dataprotection@manchester.ac.uk or write to The Information Governance Office, Christie Building, The University of Manchester, Oxford Road, M13 9PL at the University and we will guide you through the process of exercising your rights.

You also have a right to complain to the Information Commissioner's Office about complaints relating to your personal identifiable information (https://ico.org.uk/concerns) Tel 0303 123 1113.

Contact Details

If you have any queries about the study or if you are interested in taking part, then please contact a member of the site research team:

<site to insert name,

tel number and

email address of site contacts for the study. Note these must be in LARGE BOLD PRINT>

Further information about the study can also be found on the study website: https://leader.octru.ox.ac.uk

Thank you for taking the time to read this Information Sheet and considering taking part
