# Supplementary material for: Clinical and cost-effectiveness of oral versus intramuscular glucocorticoids in rheumatoid arthritis: protocol for a multicentre randomised controlled trial with economic evaluation and qualitative sub-study (LEADER trial)
Source: BMJ Open. 2026 Jul 10;16(7):e119885. doi: 10.1136/bmjopen-2026-119885 (PMC13358284; doi:10.1136/bmjopen-2026-119885)
Supplement: online supplemental file 3 [file bmjopen-16-7-s003.pdf]

The University of Manchester

To be printed on Trust/equivalent headed paper

Oral versus intramuscular glucocorticoids in rheumatoid arthritis

Consent Form

If you are happy to participate please complete and sign the consent form below

	**Activities**	Initials
1	I confirm that I have read the information sheet (**Version XX, Date dd/mon/yyyy**) for the above study and have had the opportunity to consider the information and ask questions and had these answered satisfactorily.	
2	I understand that my participation in the study is voluntary and that I am free to withdraw at any time without giving a reason and without detriment to myself. I understand that it will not be possible to remove my data from the project once it has been anonymised and forms part of the data set.<br><br>I agree to take part on this basis.	
3	I agree to my GP being informed of my participation in this study.	
4	I agree to have blood samples taken for the research purpose as explained to me.	
5	I agree that any data collected may be included in anonymous form in publications, reports, summaries of the study or at conferences.	
6	I agree that any anonymised data collected may be made available to other researchers.	

7	I understand that my full name, address, bank details and email address will be passed to the University of Oxford's Finance team for the sole purpose of sending me the financial reimbursement by BACS.	
8	I understand that the personal data as explained in the information sheet will be shared with Forth so I can complete the finger prick blood test.	
9	I understand that relevant sections of my medical notes and data collected during the study may be looked at by individuals from The University of Manchester, King's College Hospital NHS Foundation Trust, the University of Oxford, the NHS Trust/provider or regulatory authorities, where it is relevant to my taking part in this research. I give permission for these individuals to have access to my data.	
10	I understand that there may be instances where during the course of the research information is revealed which means the researchers will be obliged to break confidentiality and this has been explained in more detail in the information sheet.	
11	I agree to take part in this study.	

The following activities are optional, you may participate in the research without agreeing to the following:

12	I agree to take part in a one to one interview or focus group and, if selected, that the interview will be audio recorded and that research publications can include direct quotes of my responses in anonymous format along with my sex and age.	
13	I agree that the researchers may store my contact details securely for up to 3 years to contact me to find out about my health and rheumatoid arthritis treatment.	
14	I agree that the researchers may retain my contact details in order to provide me with a summary of the findings for this study.	

Data Protection

The personal information we collect and use to conduct this research will be processed in accordance with data protection law as explained in the Participant Information Sheet and the [Privacy Notice for Research Participants (https://documents.manchester.ac.uk/display.aspx?DocID=37095)](https://documents.manchester.ac.uk/display.aspx?DocID=37095).

__________________________ __________________________ ______________

Name of Participant Signature Date

The University of Manchester

______________________________	______________________	____________
Name of the person receiving consent	Signature	Date

[1 copy for the participant, 1 copy for the research team (original), 1 copy for the medical notes]
