# Supplementary material for: Clinical and cost-effectiveness of oral versus intramuscular glucocorticoids in rheumatoid arthritis: protocol for a multicentre randomised controlled trial with economic evaluation and qualitative sub-study (LEADER trial)
Source: BMJ Open. 2026 Jul 10;16(7):e119885. doi: 10.1136/bmjopen-2026-119885 (PMC13358284; doi:10.1136/bmjopen-2026-119885)
Supplement: Supplementary file 4 [file bmjopen-2026-119885.reviewer_comments.pdf]

PEER REVIEW HISTORY

BMJ Open publishes all reviews undertaken for accepted manuscripts. Reviewers are asked to complete a checklist review form and are provided with free text boxes to elaborate on their assessment. These free text comments are reproduced below.

ARTICLE DETAILS

Title (Provisional)

The Clinical and Cost-effectiveness of Oral Versus Intramuscular Glucocorticoids in Rheumatoid Arthritis: Protocol for a multicentre randomised controlled trial with economic evaluation and qualitative sub-study (LEADER trial)

Authors

Cook, Jonathan Alistair; Wilson, Nicky; Wilkins, Kate; Staniland, Karen; Ekang, Iniobong; Azmi, Shazli; Heaney, Liam; Gavan, Sean; Elliott, Rachel Ann; Wilson, Robert J; Calvert, Clare; Francis, Anne; Williams, Jennifer Anne Elizabeth; Turner, Anabella; Zimmermann, Alex; Richards, Duncan; Barton, Anne; Bluett, James; trial network, the LEADER

This article was not externally reviewed at BMJ Open. Protocols that have been independently assessed prior to submission to BMJ Open are usually fast-tracked to publication on the grounds that further substantial changes will not be possible. This independent assessment will usually be external, independent review for both a substantial grant award from a non-commercial or government funder and ethics approval.
